# Reinforced Bioremediation of Excessive Nitrate in Atrazine-Contaminated Soil by Biodegradable Composite Carbon Source

**DOI:** 10.3390/polym15132765

**Published:** 2023-06-21

**Authors:** Zhongchen Yang, Yanhong Lou, Hong Pan, Hui Wang, Quangang Yang, Yajie Sun, Yuping Zhuge

**Affiliations:** National Engineering Research Center for Efficient Utilization of Soil and Fertilizer Resources, College of Resources and Environment, Shandong Agricultural University, 61 Daizong Road, Tai’an 271018, China; yzcgaara@163.com (Z.Y.); yanhonglou1985@163.com (Y.L.); panhong6239@163.com (H.P.); huiwang@sdau.edu.cn (H.W.); sttzzy@sdau.edu.cn (Q.Y.); sunyajie@sdau.edu.cn (Y.S.)

**Keywords:** biodegradable polymer, bioremediation, composite carbon source, nitrate, atrazine, microbial community structure

## Abstract

Bioremediation is a good alternative to dispose of the excessive nitrate (NO_3_^−^) in soil and alleviate the secondary salinization of soil, but the presence of atrazine in soil interferes with the bioremediation process. In the present study, the biodegradable composite carbon source with different dosages was added to the atrazine-contaminated soil to intensify the bioremediation of excessive NO_3_^−^. The atrazine-contaminated soil with a 25 g/kg composite carbon source achieved the optimal NO_3_^−^ removal performance (92.10%), which was slightly higher than that with a 5 g/kg composite carbon source (86.15%) (*p* > 0.05). Unfortunately, the negative effects of the former were observed, such as the distinctly higher emissions of N_2_O, CO_2_ and a more powerful global warming potential (GWP). Microbial community analysis showed that the usage of the composite carbon source clearly decreased the richness and diversity of the microbial community, and greatly stimulated nitrogen metabolism and atrazine degradation (*p* < 0.05). To sum up, the application of a 5 g/kg composite carbon source contributed to guaranteeing bioremediation performance and reducing adverse environmental impacts at the same time.

## 1. Introduction

Under the pressure of an increasing world population, food demands and shortage of available arable land, a large amount of nitrogen fertilizer is applied to maximize crop production. Consequently, plentiful nitrogen enters the agro-ecosystem from the atmosphere, some enters the surface water and groundwater in the form of reactive nitrogen, and some returns to the atmosphere through ammonia volatilization [1]. Such a large amount of human-driven global reactive flux has caused serious environmental, such as eutrophication in surface water, increased secondary salinization and NO_3_^−^ accumulation in soil [2]. As an important nitrogen source for plant growth, NO_3_^−^ can easily migrate in soil and water, cause infantile methemoglobinemia and induce the formation of toxic substances, such as nitrite (NO_2_^−^) and nitrosamines [3]. Thus, an effective method is urgently needed to solve the problem of excessive NO_3_^−^ in soil to improve soil quality and protect human health. At present, irrigation, replacement with clean soil, chemical immobilization and electrokinetic techniques are generally utilized to deal with excessive NO_3_^−^ in soil. However, these methods are not suitable for large-scale applications because of their high energy consumption, high cost and low efficiency. In contrast, microbial remediation techniques are widely accepted because of their low input, high yield and environmental soundness. With respect to microbial remediation techniques, microbial denitrification is the most mainstream way to completely remove NO_3_^−^ with a satisfactory economy and predominant selectivity of end products [4]. With the participation of various microbial groups in soil, NO_3_^−^ is eventually reduced to N_2_, N_2_O and NH_3_, which are reused by plants and microorganisms [5,6]. Therefore, it is a good alternative to dispose of the excessive NO_3_^−^ in the soil by microbial denitrification.

Microbial denitrification in soil mainly occurs in anoxic microhabitats with enough NO_3_^−^ and available carbon and is mediated by NO_3_^−^ supply, available organic carbon, pH, temperature and microorganisms. Specifically, the readily available organic carbon in the soil is closely related to the denitrification rate, since labile carbon is the electron donor for NO_3_^−^ reduction. As one of the largest traditional agricultural countries in the world, China has a large amount of crop straw every year. In recent years, more and more crop straws are burned in the field as fertilizer after harvest to improve agricultural soil fertility and physiochemical characteristics [7]. However, the decomposition rate of returned straw is very slow due to the recalcitrant lignin. In order to reduce the recalcitrant lignin, improve resource utilization of crop straw and strengthen denitrification performance, the corncob was pretreated with dilute sodium hydroxide solution and blended with the biodegradable polymer polybutylene succinate to prepare the composite carbon source [8], which was used to reinforce bioremediation of excessive nitrate in the soil in the present study.

In order to protect plants from pests, diseases and overgrowth of weeds, pesticides are frequently applied all over the world [9]. Despite the noticeable benefits in raising production and mitigating food scarcity, the use of pesticides also poses a potential threat to soil ecology and environmental health due to their long persistence and high toxicity [10]. Atrazine was shown to be toxic to the photosystem and growth of the plants, enzymatic processes of phytoplankter and microalgae, mutagenicity, genotoxicity, defective cell division and endocrine disruption of aquatic organisms. In addition, exposure to atrazine would lead to the disruption of sustainable agricultural soil use and an increase in dysgenesis in humans. Atrazine is one of the most widely used herbicides in China, which was used in the early 1980s to control broadleaf and gramineous weeds [11,12]. In China, the annual consumption of atrazine reached a disturbing 13 million kg in 2019. What was worse, atrazine and its metabolites have been detected in soil, surface water and groundwater, causing a range of human health problems, including cancers and birth defects, after long-term exposure [13]. It is conceivable that the residual atrazine adversely affects non-targeted organisms due to its high persistence in the environment. The residual atrazine increases soil microbial biomass and microbial respiration while decreasing soil microbial diversity and soil enzyme activity. Meanwhile, atrazine can supply available carbon and nitrogen as substrates for specific microorganisms and allow the growth of a microbial population capable of atrazine metabolism. The residual atrazine in soil inevitably appends ecotoxicological effects to soil microorganisms, adversely affects soil quality [14] and interferes with metabolic activities, including bioremediation. It was reported that the compost amendments could stimulate the growth of degrading microorganisms and enhance peroxidase activity with the help of water-extractable organic matter from compost, which could not only provide an abundant carbon source for microbial growth but also promote the adsorption and degradation of pollutants on the cell surface [15]. The bio-organic fertilizer was prepared using cattle manure organic fertilizer, biochar, poly-(γ-glutamic acid) and an atrazine-degrading strain Arthrobacter sp. DNS10 successfully achieved atrazine removal due to the plentiful binding sites for atrazine and the adsorption and fixation of biochar [16]. The sugarcane filter cake promotes microbial activity and improves microbial diversity by providing a great number of nutrients and increasing the porosity and water-holding capacity of soils [17]. However, little is known about the effects of composite carbon sources on the bioremediation performance of excessive NO_3_^−^ in atrazine-contaminated soil.

In this study, microcosmic experiments were conducted to evaluate the bioremediation performance of a composite carbon source on excessive NO_3_^−^ in atrazine-contaminated soil. The main objectives of this study were (1) to investigate the effect of the dose of a composite carbon source on NO_3_^−^ removal performance and soil properties in atrazine-contaminated soil; (2) to explore the emissions of CH_4_, N_2_O, CO_2_ and their global warming potential; and (3) to elucidate the effect of composite carbon sources on microbial communities and pivotal metabolic pathways.

## 2. Materials and Methods

### 2.1. Soil Sample Collection and Preparation of Composite Carbon Source

Field sampling was conducted in Wenyang Town, Shandong Province, China (36°0′18″ N, 116°0′77″ E). After passing through a 2 mm sieve to remove debris, the collected topsoil (0–20 cm) was placed in a cool and ventilated place to air dry for physicochemical characteristics and further use. The corncob was pretreated with a dilute sodium hydroxide solution and biodegradable polymer polybutylene succinate purchased from Shenzhen Huixin Plastic Chemical Co. Ltd. (Shenzhen, China) was used for the preparation of composite carbon source [8]. In order to mix thoroughly with the soil, the composite carbon source was ground and sieved (2 mm). Atrazine (purity > 98%) was purchased from Aladdin Reagent Co., Ltd., Shanghai, China, and its stock solution was prepared and stored in the dark at 4 °C.

### 2.2. Experimental Design

The microcosmic experiment was designed to explore the effects of doses of biodegradable composite carbon sources (three levels: 0, 5 and 25 g/kg, named C0, C5 and C25, respectively) on NO_3_^−^ removal performance in atrazine-contaminated soil. Before the formal experiment, the moisture content of the soil was regulated to 60% of the field water-holding capacity and preincubated in a thermostatic incubator (GXZ-436, Ningbo, China) at 25 °C in the dark for 1 week. To stimulate the actual application, 3 mg/kg atrazine at the recommended dose was selected in this study [18]. Every 50 g atrazine-treated soil, composite carbon source (three levels: 0, 5 and 25 g/kg) and 4 mL KNO_3_ solution (1.5 mg/mL NO_3_^−^-N) were fed to 250 mL serum bottles, respectively. Each serum bottle was injected with enough N_2_ to remove oxygen and was sealed with a rubber plug. Each treatment was carried out in triplicate. All of the soil samples were incubated in an incubator (GXZ-436, Ningbo, China) at 25 °C in the dark. During the experiment, distilled water was supplemented to sustain stable soil water content (60% of the field water-holding capacity). The destructive sampling strategy was performed on days 0, 7, 14, 21, 28, 35 and 42, and obtained a total of 63 samples (3 treatments × 3 replicates × 7 sampling time). The NO_3_^−^ and NH_4_^+^ contents in the soil samples were extracted with 1 M KCl, and measured using a flow injection analyzer (SEAL Auto Analyzer AA3, Norderstedt, Germany). At the end of the experiment, 20 mL of gas samples were collected from each sealed serum bottle using a syringe, and the concentrations of CH_4_, N_2_O and CO_2_ were determined using a gas chromatograph (Shimadzu GC-2010 Plus, Kyoto, Japan). The soil pH was measured using potentiometry with a soil-to-water ratio of 1:2.5. Soil dissolved organic carbon content was determined using a TOC analyzer (Shimadzu TOC-C VPN 200 V, Kyoto, Japan).

### 2.3. Soil DNA Extraction and Illumina MiSeq Sequencing Analysis

Total microbial genomic DNA was extracted using a microbial DNA extraction kit (Biocolors, Shanghai, China) according to the manufacturer’s instructions, and the amount of extracted DNA was determined using NanoDrop ND-2000 (Nanodrop Technologies, Wilmington, NC, USA). To investigate the effects of the composite carbon source on microbial community structure, the bacterial 16S rRNA gene (V3-V4 regions) and fungal internal transcribed spacer (ITS1 region) were amplified using primers 338F/806R and ITS1F/ITS2R, respectively. High-throughput sequencing of the bacterial 16S rRNA and fungal ITS rRNA genes was conducted using the Illumina MiSeq PE300 platform (Illumina, San Diego, CA, USA). After removing the ambiguous, homologous and short sequences, the high-quality sequences were clustered into operational taxonomy units (OTUs) at a similarity threshold of 97% [19]. Phylogenetic investigation of communities by reconstruction of unobserved states (PICRUSt) was conducted to establish a linkage between the structure of the microbial community and microbial functions [20].

### 2.4. Statistical Analysis

The calculations of average and standard deviation were conducted using Excel 2019 (Microsoft 2019). Differences between groups regarding the concentrations of NO_3_^−^-N and NH_4_^+^-N, soil pH and dissolved organic carbon, greenhouse gas emissions, Ace index, Shannon index and functional pathways were analyzed by one-way ANOVA (*p* < 0.05) using SPSS 26.0 software (IBM, Chicago, IL, USA). Considering the different warming potentials of greenhouse gases, the global warming potentials of different treatment groups were calculated to evaluate the possible warming effects using the radiative forcing potential of 298 for N_2_O and 25 for CH_4_ relative to CO_2_. Partial least-square discriminant analysis (PLS-DA) was performed to evaluate the variations in the bacterial and fungal communities among the different treatments.

## 3. Results and Discussion

### 3.1. Effect of Composite Carbon Source on Soil Denitrification Performance

**Figure 1:**
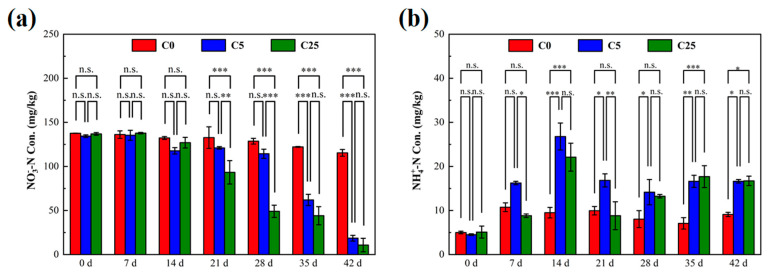
The concentrations of NO_3_^−^−N (**a**) and NH_4_^+^−N (**b**) in soils with different treatments. The symbol *, ** and *** mean that the correlation is statistically significant at the 0.05, 0.01 and 0.001 levels, respectively.

The variations of NO_3_^−^-N and NH_4_^+^-N in the soils are shown in Figure 1. The initial NO_3_^−^-N concentrations in C0, C5 and C25 were 137.61 mg/kg, 134.36 mg/kg and 136.91 mg/kg, respectively. After 21 days of incubation, lower NO_3_^−^-N contents were evident in C5 and C25 than in C0 (*p* < 0.01). The early delayed denitrification performance in C5 and C25 might be caused by the toxic effect of atrazine on denitrifying microorganisms, the microbial adaptation period before biodegradation of atrazine, microbial attachment and decomposition of the carbon source [21]. At the end of the incubation, the minimum NO_3_^−^-N concentration was determined in C25 (10.81 mg/kg) with a NO_3_^−^-N removal efficiency of 92.10%, which was slightly lower than C5 (18.61 mg/kg) (*p* > 0.05). These results demonstrated that the addition of a composite carbon source clearly promoted the removal of NO_3_^−^-N from atrazine-contaminated soil. Soil organic carbon was reported to be the primary soil property associated with denitrification, in which it serves as an electron donor and converts NO_3_^−^-N to N_2_O or N_2_ [22,23]. The composite carbon source could provide a large number of electron donors for denitrification microorganisms, which was conducive to accelerating the soil denitrification rate [24].

The NH_4_^+^-N concentration was 4.87 ± 0.31 mg/kg before the incubation. Compared with the initial stage of incubation, NH_4_^+^-N contents in all treatment groups increased significantly, especially in C5 (16.63 mg/kg) and C25 (16.73 mg/kg). The significant increase in NH_4_^+^-N content could be attributed to two factors: the mineralization of atrazine and dissimilatory nitrate reduction to ammonium. The mineralization of atrazine conducted by indigenous microorganisms predominated in C0 due to the lack of carbon sources, and the generated cyanuric acid could be further mineralized to NH_4_^+^-N. With the addition of the composite carbon source in C5 and C25, more small molecular substrates were produced, which increased the ratio of carbon to NO_3_^−^ (C/NO_3_^−^) and stimulated soil dissimilatory nitrate reduction to ammonium [6]. Hence, more NH_4_^+^-N was accumulated in C5 and C25. Anaerobic conditions are favorable for denitrification, but not for the biodegradation of atrazine, in which oxygen acts as an electron acceptor and atrazine serves as an electron donor [25]. However, highly reactive microbial activity was obtained after the addition of a composite carbon source [26].

### 3.2. Changes of pH and Dissolved Organic Carbon in Different Treatments

**Figure 2:**
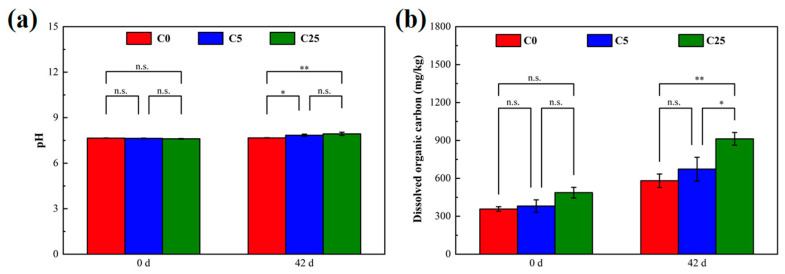
The soil pH (**a**) and dissolved organic carbon (**b**) in different treatments. The symbol * and ** mean that the correlation is statistically significant at 0.05 and 0.01, respectively.

Figure 2 illustrates the changes in the soil pH and dissolved organic carbon at the beginning and end of incubation. The soil pH values at C5 and C25 significantly elevated, whereas an obvious increase in dissolved organic carbon occurred only at C25. The pH and dissolved organic carbon are not only important factors regulating soil denitrification but also mediate the degradation of herbicides [22,27,28]. Although the pH increased after the addition of carbon sources, it remained in the near-neutral range, which was conducive to creating a microenvironment suitable for microbial survival, maintaining microbial activity, denitrification and atrazine mineralization [3,29]. On the one hand, dissolved organic carbon is used as an electron donor and energy source to sustain microbial growth, and ultimately reduce NO_3_^−^-N to N_2_ in biological denitrification [30]. On the other hand, dissolved organic carbon could stimulate the biodegradation of herbicides by increasing microbial populations and activities [31,32]. In general, the composite carbon source played an indispensable role in strengthening microbial denitrification and atrazine degradation.

### 3.3. Greenhouse Gas Emissions and Their Global Warming Potential

**Figure 3:**
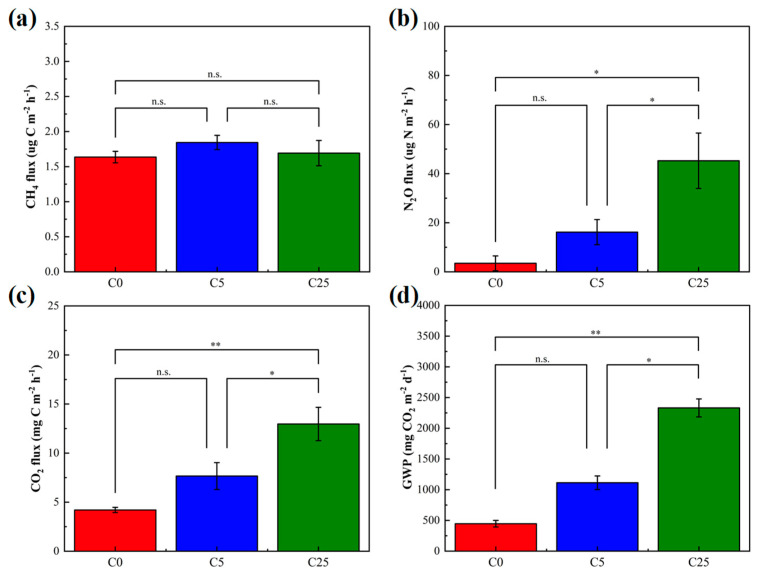
The soil greenhouse gas mean fluxes of CH_4_ (**a**), N_2_O (**b**), CO_2_ (**c**) and their global warming potential (GWP) (**d**). The symbol * and ** mean that the correlation is statistically significant at 0.05 and 0.01, respectively.

CH_4_, N_2_O and CO_2_ are important components of greenhouse gases. Among the greenhouse gases measured, there was no significant difference in CH_4_ flux, whereas the mean fluxes of N_2_O and CO_2_ in C25 were distinctly enhanced than those in the C0 and C5 treatments (Figure 3). The mean fluxes of CH_4_ in C5 and C25 treatments were slightly higher than those in C0 (*p* > 0.05). CH_4_ is generally generated by methanogenic bacteria during the anaerobic digestion of organic matter and is eliminated by microbial oxidation in soils [33]. The anaerobic microenvironment was probably the main reason for this result. Generally, N_2_O emissions from soil are regulated by microbial nitrification and denitrification [34], and most N_2_O comes from microbial denitrification in an anaerobic microenvironment. The accessible carbon sources, NO_3_^−^-N contents, temperature and pH are important factors determining the amount of N_2_O emissions [35]. The number of available carbon sources in C25 was higher than that in C0 and C5 treatments, but the N_2_O reduction was dramatically hindered. The supplementation with easily mineralizable carbon from a composite carbon source stimulates microbial denitrification and increases N_2_O fluxes [36]. Previous studies reported that the organic substrates added to soil could increase microbial biomass, stimulate metabolic activities, including soil mineralization, promote the conversion of soil organic carbon and eventually lead to an increase in CO_2_ emissions [37,38]. It has been widely demonstrated that the straw return provides more available carbon and nitrogen for microbes and thus stimulates N_2_O and CH_4_ emissions [39,40]. Not only that but the changes in soil physical and chemical properties due to straw return are also non-negligible factors affecting greenhouse gas emissions. The GWP in C25 was largely higher than that of C0 and C5 (5.22 times and 2.09 times, respectively) due to the high CO_2_ fluxes, which suggested the risk of significantly increasing greenhouse potential in C25 (*p* < 0.01). Similarly to straw return, the use of a composite carbon source increases the greenhouse effect potential to a certain extent. The soil micro-organisms gathered and reproduced in large numbers around the composite carbon source, which accelerated the decomposition process, released a large number of organic nutrients, increased organic content in the soil and facilitated the flow of nitrogen in microorganisms. The application of a composite carbon source may affect greenhouse gas emissions by changing the storage effect of carbon and nitrogen, eventually influencing the GWP [41].

### 3.4. Microbial Community Structure

#### 3.4.1. Composition and Diversity of Soil Bacterial and Fungal Community

Illumina MiSeq sequencing was used to investigate the diversity and structure of the microbial communities; there were 352,863 and 395,002 effective sequences with an average length of 419.23 bp and 247.76 bp for the soil bacterial and fungal communities, respectively. The optimized sequences were clustered into 2423 OTUs of bacteria and 1066 OTUs of fungi with a threshold value of 0.97. There were 1599, 1157 and 1270 bacterial OTU numbers in C0, C5 and C25 treated soils, respectively, while those of fungal OTU numbers were 408, 203 and 239 in C0, C5 and C25 treated soils, respectively. These results showed that both bacterial and fungal OTU numbers in C5 and C25 treated soils were significantly lower than those in C0 soils, suggesting that the microbial community diversity, to some extent, was decreased by the addition of the composite carbon source.

**Figure 4:**
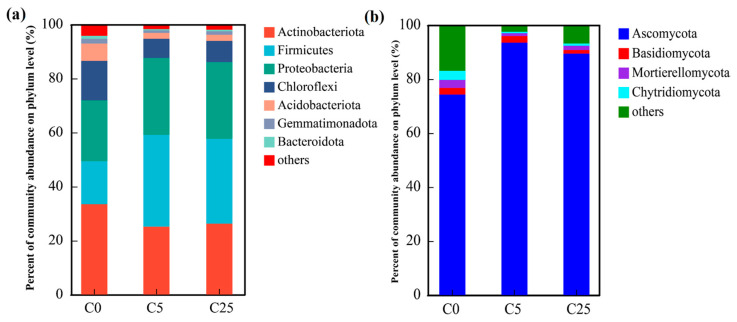
The compositions of soil bacterial communities (**a**) and fungal communities (**b**) at the phylum level.

The dominant bacterial phyla in the soil samples (relative abundance > 1.00%) included Actinobacteriota, Firmicutes, Proteobacteria, Chloroflexi, Acidobacteriota, Gemmatimonadota and Bacteroidota (Figure 4a). These phyla accounted for over 95% of the bacterial sequences. The relative abundance of Actinobacteriota was significantly decreased from 33.49% in C0 to 25.16% and 26.29% in C5 and C25, respectively. In addition, the relative abundance of Firmicutes and Proteobacteria appreciably increased from 15.89% and 22.59% in C0 to 31.40–34.00% and 28.40–28.46% in C5 and C25, respectively. Compared with C0, the relative abundances of the remaining major phyla in the C5 and C25 treatments were significantly reduced. The differences in soil microbial communities could be largely due to the application of a composite carbon source, which supported eutrophic ecosystems and enriched the copiotrophic taxa rather than oligotrophic ecosystems. Chloroflexi could participate in nitrate reduction and nitrite oxidation in the soil and promote the transformation of soil nitrogen [42]. Acidobacteria and Proteobacteria play a significant role in the soil nitrogen and carbon cycles [43]. Bacteroidetes are responsible for the decomposition of soil organic matter into small-molecule organic carbon [42].

Regarding the fungal communities (Figure 4b), only four primary phyla (relative abundance > 1.00%) were observed in all samples. Ascomycota achieved an enormous numerical advantage (74.28–93.51%) over Basidiomycota (1.30–2.47%), Mortierelleomycota (1.07–2.92%) and Chytridiomycota (0.56–3.37%). The relative abundance of Ascomycota in C5 and C25 was evidently boosted by the input of the composite carbon source, accompanied by a sharp decline in the relative abundance of other fungal phyla, which is consistent with the results obtained by Li et al. [44].

Pesticides are typically toxic substances that have detrimental effects on sensitive members of the soil microbial community. After the application of atrazine, the number of species that can tolerate atrazine toxicity and metabolize atrazine increased, whereas the number of sensitive species decreased, resulting in changes in the soil microbial community. In addition, as a biodegradable material, the composite carbon source could be degraded by a few microorganisms and enzymes attached to its surface, generating large amounts of small molecule organic matter, which can be easily utilized by colonized microorganisms. In this situation, the degrading microorganisms of the composite carbon source will gain nutritional advantages, which may affect the community structure to some extent.

**Figure 5:**
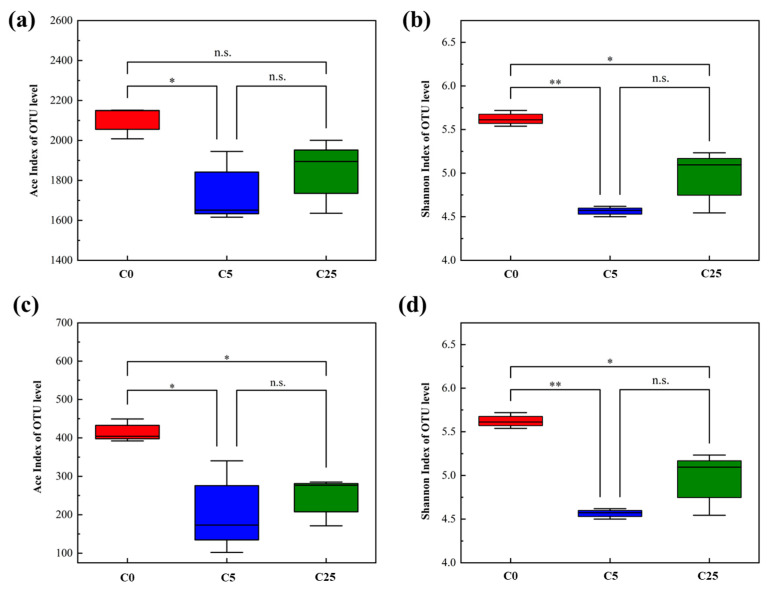
Diversity of soil microbial community. Ace index (**a**) and Shannon index (**b**) of bacterial communities; Ace index (**c**) and Shannon index (**d**) of fungal communities. The symbol * and ** mean that the correlation is statistically significant at the 0.05 and 0.01 levels, respectively.

The diversity indexes (Ace index and Shannon index) of the soil microbial community are presented in Figure 5. The results showed that the addition of the composite carbon source decreased the richness and diversity of the bacterial community, and the decreasing trend was more obvious in atrazine-contaminated soil with a 5 g/kg composite carbon source (*p* < 0.05). Likewise, the richness and diversity of the fungal community in the composite carbon source with atrazine-contaminated soil exhibited a distinct downward trend (*p* < 0.05).

**Figure 6:**
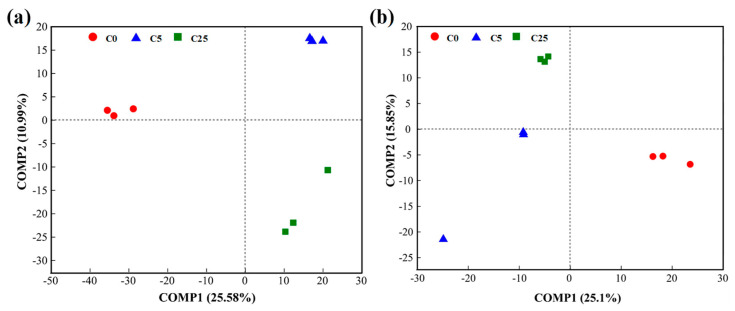
PLS−DA of bacterial (**a**) and fungal (**b**) communities in soils.

The PLS-DA analysis showed that both bacterial and fungal communities under different treatments were well separated along the COMP1 axis (25.58% and 25.10%, respectively) (Figure 6). These results implied that the application of a composite carbon source visibly changed the compositions of the bacterial and fungal communities.

#### 3.4.2. Effect of Composite Carbon Source on Microbial Functions

**Figure 7:**
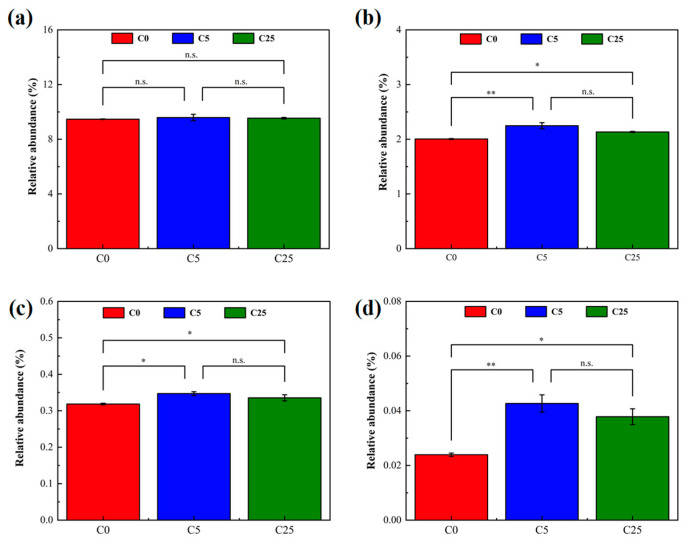
The relative abundance of metabolic pathways predicted by PICRUSt2. (**a**) Carbohydrate metabolism; (**b**) xenobiotic biodegradation and metabolism; (**c**) nitrogen metabolism; (**d**) atrazine degradation. The symbol * and ** mean that the correlation is statistically significant at 0.05 and 0.01, respectively.

The relative abundance of carbohydrate metabolism, xenobiotic biodegradation and metabolism at pathway level 2, and nitrogen metabolism and atrazine degradation at pathway level 3 were selected from PICRUSt2 to analyze the effect of the composite carbon source on microbial functions (Figure 7). The input of the composite carbon source did not significantly alter the microbes’ carbohydrate metabolism, but promoted xenobiotic biodegradation and metabolism (*p* < 0.05), and stimulate nitrogen metabolism (*p* < 0.05) and atrazine degradation (*p* < 0.05). Atrazine is toxic to microorganisms exposed to the environment, affecting soil enzyme activity and microbial diversity [45]. The introduction of a composite carbon source increased the microbial metabolic activity, stimulated the degradation ability of indigenous microorganisms and realized the simultaneous removal of NO_3_^−^-N and atrazine, which effectively alleviated soil acidification and the toxic effect of atrazine.

## 4. Conclusions

The composite carbon source obviously promoted NO_3_^−^-N removal and NH_4_^+^-N accumulation in atrazine-contaminated soil. Compared to atrazine-contaminated soil with high doses of composite carbon source, the soil with 5 g/kg of composite carbon source obtained comparable NO_3_^−^-N removal performance with fewer emissions of greenhouse gases and lower GWP, which might be a more suitable approach for the bioremediation of excessive NO_3_^−^-N in atrazine-contaminated soil. The application of the composite carbon source largely decreased the richness and diversity of the microbial community, visibly changing the compositions of the bacterial and fungal communities. In addition to carbohydrate metabolism, xenobiotic biodegradation and metabolism, nitrogen metabolism and atrazine degradation were significantly stimulated by the composite carbon source.

## Data Availability

Data are available upon request due to privacy and ethical restrictions.
